# Retrospective Analysis of Treatment Patterns in Pseudophakic Diabetic Macular Oedema Eyes Treated with Anti-VEGF

**DOI:** 10.1155/2021/9967831

**Published:** 2021-07-27

**Authors:** Di Zou, Imran Jawaid, Winfried M. Amoaku

**Affiliations:** ^1^Queen's Medical Centre, Derby Road, Nottingham NG72UH, UK; ^2^Division of Clinical Neurosciences, Academic Ophthalmology, University of Nottingham, Nottingham University Hospitals NHS Trust, Nottingham, UK

## Abstract

**Methods:**

We performed a retrospective review of outcomes in 81 pseudophakic eyes with DMO that received at least 6 anti-VEGF injections. We reviewed baseline and posttreatment optical coherence tomography images, visual acuity, prescribing patterns, time taken to deliver anti-VEGF injections, and structural and functional outcomes.

**Results:**

It took an average of 913 ± 454.1 days to deliver a mean of 11.1 ± 4.7 anti-VEGF injections. Time from baseline to receiving the first 6 anti-VEGF injections was longer than 9 months in 74.7% (*n* = 59/79) of eyes. There was a mean gain of 1.6 letters (−0.03 logMAR) from baseline to the end point. After 5 anti-VEGF intravitreal injections, the mean CMT was 391.9 *μ*m from 474.4 *μ*m at baseline (*p* < 0.0001). In 52 of 79 eyes (65.8%), more than one type of anti-VEGF agent was used.

**Conclusions:**

The anti-VEGF treatment used to treat these eyes with DMO was suboptimal, a finding consistent with recently published “real-world” data. There was a strong tendency for patients to be switched within the class to a second anti-VEGF agent.

## 1. Introduction

In the UK, the prevalence of diabetes has increased from an estimated 2.8% in 1996 to 4.3% in 2005—an increase of more than 50% in 10 years [1]. In the next 20 years, it is projected that the population with diabetic retinopathy (DR) will increase by at least 20%, assuming age-specific prevalence rates remain constant. However, if prevalence rates increase in line with other Western countries, an increase of between 50 and 80% is projected [1]. It is anticipated that these changes will lead to a decline in the quality of life of patients along with greater morbidity and increased health care resource utilisation [1, 2].

Diabetic macular oedema (DMO) is a common, specific form of DR, which results from the accumulation of fluid into, and thickening of, the macula; it is one of the most common causes of vision loss in patients with diabetes [3]. Current therapeutic options for DMO in the UK include focal/grid laser photocoagulation; intravitreal injections (IVTs) of antivascular endothelial growth factor (anti-VEGF) drugs, including ranibizumab (Lucentis™; Genentech, San Francisco, CA, USA), aflibercept (EYLEA™; Regeneron Pharmaceuticals, Tarrytown, NY, USA), and off-label bevacizumab (Avastin™; Genentech, San Francisco, CA, USA); as well as intravitreal corticosteroids, including the dexamethasone implant (OZURDEX® 700 *μ*g; Allergan Ltd., Marlow, Buckinghamshire, UK) and fluocinolone acetonide implant (ILUVIEN® 190 *μ*g; Alimera Sciences Ltd., Aldershot, Hampshire, UK).

In England, the National Institute for Health and Care Excellence (NICE) approved anti-VEGF (ranibizumab and aflibercept) as a first-line treatment in patients with DMO provided the eye had a central retinal thickness (CRT) of ≥400 microns (as measured on optical coherence tomography (OCT)) at the start of treatment [4, 5]. However, NICE does not recommend anti-VEGF therapy for DMO where the CRT is <400 microns, as they are not considered to be cost-effective [4, 5]. Intraocular corticosteroids (dexamethasone and fluocinolone acetonide implants) are used as a second-line therapy. NICE has approved their use only in eyes with a pseudophakic lens and inadequate response to prior therapy/noncorticosteroid treatments, or where such treatment is unsuitable [6, 7].

An increase in retinal thickness is linked to reduced visual function and is, therefore, a good indicator of vision loss in patients with DMO. However, there may be a disconnect between the functional changes (as measured by visual acuity) and morphological changes (as measured by retinal thickness) on OCT. Furthermore, OCT measurements are more objective reproducible measurements than VA measured in a routine clinical setup. As such, investigating visual acuity alongside the change in CRT is necessary to confirm the effectiveness of therapies [8, 9].

The effectiveness and safety of anti-VEGF therapies in DMO have been reported in several studies, including the RESTORE [10], RIDE and RISE [11], VIVID and VISTA [12], and BOLT trials [13]. More recently, Protocol T, reported by the Diabetic Retinopathy Clinical Research Network (DRCR.net), compared the effects of aflibercept, bevacizumab, and ranibizumab after two years of therapy. This study reported that in patients with DMO, on average, little difference was seen in VA gain at year two between aflibercept and ranibizumab, although the outcomes were superior to bevacizumab [14].

In cases where the initial anti-VEGF therapy was inadequate in treating DMO, studies have been conducted to investigate the effects of switching within the anti-VEGF class [15–17]. Between these studies, the reasons for switching varied but included differences in the affinity for VEGF-A, as is the case for aflibercept that reportedly has a greater affinity for VEGF-A compared with ranibizumab and bevacizumab [18]. Other reasons included the half-life of the drug injected, for example, aflibercept is a larger molecule and has a longer half-life [19], and, lastly, tachyphylaxis, where it has been postulated that switching anti-VEGF agents could overcome this [20]. In some cases, it is apparent that some patients respond well to the change in anti-VEGF therapy, but it is not known whether it has beneficial functional and anatomical effects, or whether the observed response relates to the accumulation of injections rather than the actual change in anti-VEGF agent [17]. To date, there are no randomised control trials to determine the benefits of switching from one anti-VEGF to another in patients with DMO [21].

Even with rigorous anti-VEGF therapy, however, nearly half of all patients with DMO experience suboptimal responses as shown by persistent macular thickening and/or only moderate improvements in visual acuity [12, 22–25]. Indeed, suboptimal responses to ranibizumab were reported by Gonzalez et al. in around 40% of patients with DMO; these effects were predicted after just 3 to 6 IVTs [26]. This is indicative of the need to identify alternative treatment options to reduce the overall disease and treatment burden for patients with DMO and the physicians treating them [22, 26–28].

As pseudophakic eyes with DMO unresponsive to treatments with anti-VEGF therapies qualify for switching to other therapies including intravitreal corticosteroids (as recommended by NICE TAs [4–7]), such eyes offer the best opportunity to investigate intravitreal therapeutic patterns in the treatment of DMO in a routine clinical setting.

Here, we identified a cohort of pseudophakic patients that had been receiving anti-VEGF therapy for DMO. The objective was to understand structural and functional outcomes and examine the detail of their anti-VEGF treatment delivery, including time taken to deliver 6 anti-VEGF and the description of anti-VEGF therapies received (e.g., the frequency of within class switching).

## 2. Materials/Subjects and Methods

### 2.1. Study Design

This retrospective study utilised Medisoft® (Medisoft Ltd., Leeds, UK) to identify the electronic medical records (EMRs) of patients at the Queens Medical Centre in Nottingham, UK, that received intravitreal therapies for the treatment of DMO. Ethics approval was not required as this study was a service evaluation.

Patient selection was based on three criteria: (1) the diagnosis of DMO, with other indications and copathologies ruled-out during EMR review; (2) having received prior treatment with ≥6 IVTs of anti-VEGF (bevacizumab, ranibizumab, or aflibercept), and (3) the presence of a pseudophakic lens or was planned imminently, which would qualify patients for all NICE-approved DMO therapies [4–7]. All treatments had been carried out at the treating clinicians' discretion. Using the above approach, 79 patient eyes were identified. Patient data were pseudoanonymised and then amalgamated into a single data set.

Images of the OCT were collected using Heidelberg Spectralis and/or Topcon machines. In the majority of cases, the same machine was used for each patient. Best-recorded visual acuity (BRVA) was collected using logMAR charts and converted to ETDRS letters according to Gregori et al. [29]. The BRVA represents the best acuity with the patient's distance correction with pinhole as necessary. OCT parameters measured included CMT (microns), maximal macular thickness (MMT; microns), and foveal status (i.e., individual OCTs were read and classified as “dry” where a normal foveal contour was observed in the absence of oedema).

To be included in this analysis, all parameters needed to have been recorded at baseline (i.e., the first assessments post-DMO diagnosis/the reading prior to first IVT of anti-VEGF), and then BRVA outcomes were reported after 6 anti-VEGF injections. The morphological (OCT) outcomes were reported after the fifth initiating dose of anti-VEGF (OCT outcomes were defined after 5 IVTs as this was the next available time point where OCTs were available from initiation of therapy for this cohort (reflecting local practice)). For CMT and MMT, 78 of 79 patient eyes were included and one case was removed because the baseline value was missing. For BRVA, 76 of 79 cases were included and 2 cases were excluded due to lack of true (before anti-VEGF) baseline reading and another case was removed as the posttreatment outcome reading was reported after only 3 IVTs. Furthermore, in 3 of 76 cases, the BRVA outcomes were reported after 7 IVTs of anti-VEGF.

### 2.2. Data Collection

Data were collected to enable treatment patterns to be defined along with the time taken to deliver IVTs of anti-VEGF and associated clinical outcomes. Specific analyses included the following:

1. The time taken to deliver the first 6 IVTs of anti-VEGF
2. The overall rate of anti-VEGF treatment (for all and by anti-VEGF class) was calculated as (i) the mean time to deliver a mean number of anti-VEGF injections and (ii) the mean IVT rate for the first 6 anti-VEGF treatments (expressed as 1 IVT per number of days)
3. Quantification of the extent of switching between anti-VEGF agents
4. BRVA outcomes after 6 anti-VEGF injections and reported as mean gains or losses, maintenance (±4 letters), and improvements or losses of 5–9 letters, 10–14 letters, and ≥15 letters from baseline values
5. BRVA outcomes based on maintenance or achievement of driving vision (taken as ≥70 letters)
6. CMT and MMT outcomes after 5 anti-VEGF injections in terms of absolute changes, a change of ≥50 microns, an anatomical response (defined as a reduction of ≥20% from baseline [26, 30, 31]), and the proportion of patients achieving ≤250 microns, ≤300 microns, and ≤400 microns
7. Drying of the fovea (as discussed previously)

### 2.3. Data and Statistical Analysis

Data are presented as mean ± standard deviation or as a percentage representing the proportion of patients. Statistical analyses were conducted using the Student's paired *t*-tests to compare mean values after intervention with baseline values. Statistical significance was taken as a *p* value ≤0.05.

## 3. Results

Overall, 79 eyes were identified with DMO, with a male predominance (53 eyes vs. 26 female eyes) and a mean age of 71 years (range 44–89 years; Table 1). Of the 79 total eyes, 38 right and 41 left eyes were treated; 77 eyes were pseudophakic with 2 cases awaiting cataract surgery at baseline (Table 1). All 79 eyes had been previously treated with IVT of anti-VEGF therapies, with a mean of 11.1 ± 4.7 injections per eye, ranging between 6 and 22 injections over a mean of 913 ± 454.1 days (Table 1). Of the total 876 IVT anti-VEGF injections recorded, with patients having mainly received ranibizumab (467 injections in 68 eyes), followed by aflibercept (391 in 59 eyes), and bevacizumab (19 in 7 eyes; Table 1), 28% of eyes received 10–14 anti-VEGF injections, while 20% of eyes received ≥15 anti-VEGF injections.

### 3.1. Switching between Anti-VEGF Treatment Agents

In 52 of 79 eyes (65.8%), more than one type of anti-VEGF agent was used. Of these, ranibizumab was used as the first-line therapy in 90.4% of eyes (i.e., 47 of 52 eyes); aflibercept and bevacizumab were used as the first-line treatment in 2 and 3 eyes, respectively. A total of 3 eyes had been treated with all 3 anti-VEGF agents.

### 3.2. Time Taken to Deliver 6 IVTs of Anti-VEGF

**Figure 1:**
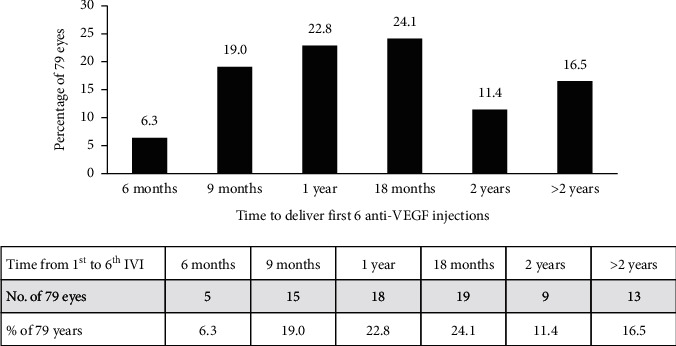
Time taken to deliver ≥6 anti-VEGF intravitreal injections. Notes: IVI: intravitreal injection; Mt: month; VEGF: vascular endothelial growth factor; Yr: year.

Overall, 74.7% (*n* = 59/79) of eyes took longer than 9 months to receive their first 6 anti-VEGF injections. Around 51.9% (*n* = 41/79) of eyes waited for more than a year to receive 6 anti-VEGF injections, with 16.5% of eyes waiting for ≥2 years (Figure 1).

### 3.3. Overall Rate of Anti-VEGF Treatment

It took an average of 913 ± 454.1 days to deliver a mean of 11.1 ± 4.7 anti-VEGF injections (Table 2). The mean injection rate for the first 6 anti-VEGF injections was 1 injection every 77.8 ± 45.0 days. The mean injection rate for all injections delivered was 1 injection every 83.9 ± 35.4 days. A subanalysis was conducted to assess the mean time to 6 injections for patients who received aflibercept or ranibizumab as their first therapy. This demonstrated that both mean time to 6 injections (328.4 ± 110.0 days vs. 480.5 ± 260.8 days) and injection rate (54.7 ± 18.3 days vs. 80.1 ± 43.5 days) were slowed for eyes who received ranibizumab as their first 6 anti-VEGF treatments (Table 2).

### 3.4. BRVA Outcomes after 6 Anti-VEGF Injections

BRVA outcomes were presented for 76 of 79 eyes. Two cases were removed due to lack of true baseline (pre-IVT) values and 1 case was removed as posttreatment outcome value was available only after 3 injections. BRVA was divided into three groups (≤0.3 logMAR (<55 ETDRS letters), 0.3 to ≤0.6 logMAR (≥55 and < 70 letters), and >0.6 logMAR (≥70 letters)).

**Figure 2:**
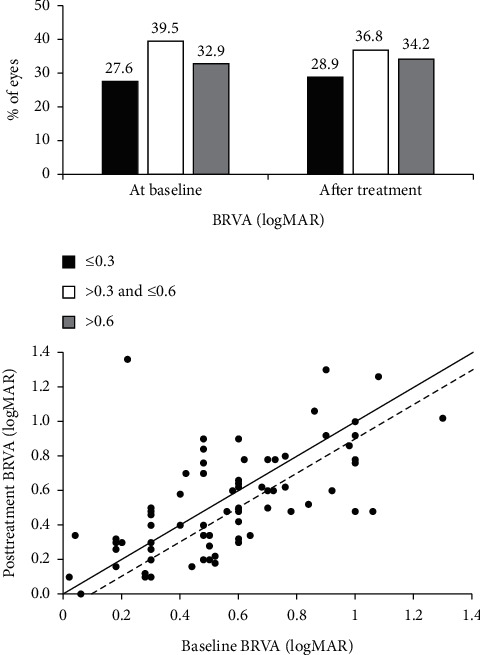
Best-recorded visual acuity at baseline and after 6 intravitreal anti-VEGF injections. Notes: the solid line represents the 45-degree line, and the dashed line represents a 0.1 logMAR (5 letters) change from the 45-degree line following treatment. Mean baseline BRVA was 0.55 ± 0.27 logMAR (mean ± SD), and there was a nonsignificant improvement (*P* = 0.269) to 0.52 ± 0.30 logMAR after anti-VEGF treatment. Black filled bars show BRVA ≤0.3 logMAR, open bars >0.03 and ≤ 0.6 logMAR, and grey filled bars >0.06 logMAR.

After IVT of anti-VEGF, the percentage of patients with a BRVA between 0.3 and ≤0.6 logMAR decreased slightly to 36.8% from a baseline of 39.5%. The opposite was true for the other two groups where slight increases were observed: >0.6 logMAR group, increased to 34.2% from a baseline of 32.9%, and ≤0.3 logMAR group, increased to 28.9% from a baseline of 27.6% (Figure 2). Overall, after anti-VEGF therapy, there was a mean gain of 1.6 letters (−0.03 logMAR) from baseline.

VA was maintained (±4 letters from baseline values) in 30.3% of eyes, while 43.4% experienced an improvement in VA (17.1% gained 5–9 letters, 11.8% gained 10–14 letters, and 14.5% gained ≥15 letters) and 26.3% of eyes experienced a loss in VA (11.8% lost 5–9 letters, 6.6% lost 10–14 letters, and 7.9% lost ≥15 letters) after anti-VEGF therapy.

Best-recorded visual acuity outcomes based on driving vision (i.e., 70 letters).

- Baseline VA ≥70 letters (≤0.3 logMAR; *n* = 21 eyes): following anti-VEGF therapy, mean VA changed from 0.23 ± 0.09 logMAR (median 0.28) to 0.32 ± 0.27 logMAR (median 0.30), which represents a mean loss of 4.4 letters from baseline. Of the 21 eyes, 14 maintained driving vision following therapy.
- In this subgroup, the highest (42.9% (*n* = 9)) proportion of patients lost VA (i.e., ≥5 letters), followed by 38.1% (*n* = 8) that maintained VA (±4 letters) and 19.0% (*n* = 4) of eyes that gained vision (≥5 letters).
- Baseline VA <70 letters (>0.3 logMAR; *n* = 55 eyes): in contrast to patients with a baseline VA ≥70 letters, anti-VEGF therapy led to mean VA gain of 3.9 ETDRS (i.e., from 0.68 ± 0.21 logMAR at baseline to 0.60 ± 0.27 logMAR. However, fewer eyes (8 of 55; 14.5%) achieved driving vision.
- In this grouping, the majority of eyes (52.7% (*n* = 29)) gained VA followed by 27.3% (*n* = 15) maintaining VA and 20.0% (*n* = 11) losing VA.

### 3.5. Central Macular Thickness Outcomes after 5 Anti-VEGF Injections

**Figure 3:**
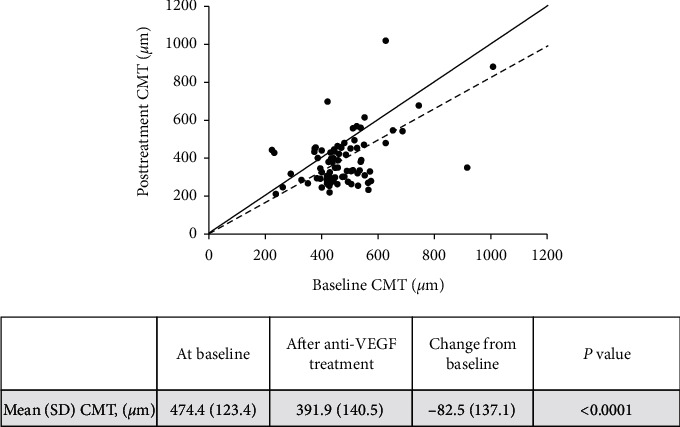
Central macular thickness at baseline and after 5 anti-VEGF injections. Notes: the solid line represents the 45-degree line, and the dashed line represents a 20% change from the 45-degree line following treatment.

After 5 IVTs of anti-VEGF, the mean CMT was 391.9 *μ*m from a baseline of 474.4 *μ*m, a statistically significant difference (*p* < 0.0001; Figure 3). Of the 78 reported eyes (1 eye excluded due to a missing baseline CMT value), 61.5% (*n* = 48) had a reduction of 50 *μ*m from baseline, and 48.7% (*n* = 38) had a sufficient anatomical response (reduction of ≥20% from baseline). Only 6.4% of eyes had a CMT of ≤250 *μ*m after IVT of anti-VEGF.

### 3.6. Maximal Macular Thickness Outcomes after 5 Anti-VEGF Injections

Similarly, the mean change in MMT was reduced from a baseline of 496.3 *μ*m to 435.9 *μ*m after 5 anti-VEGF injections (*p* < 0.0001; Supplemental Figure S1). A reduction of 50 *μ*m in MMT from baseline was achieved in 52.6% of eyes (*n* = 41/78 eyes), and 32.1% (*n* = 25/78 eyes) experienced a reduction of ≥20% MMT from baseline. None had a reduction in MMT to ≤250 *μ*m after anti-VEGF treatment.

### 3.7. Anatomical Response (Defined by Experiencing a ≥20% Reduction from Baseline)

After 5 IVTs of anti-VEGFs, only 48.7% (*n* = 38/78 eyes) showed an anatomic response based on a ≥20% MMT reduction from baseline. Only 6.4% (*n* = 5/78) of eyes had a reduction in CMT to ≤250 *μ*m (Supplemental Table S1).

Similarly, posttreatment, only 32.1% (*n* = 25/78) were anatomical responders with a reduction of ≥20% in MMT from baseline (i.e., less than a third were anatomically responsive even after 5 IVTs of anti-VEGF; Supplemental Table S1), and none of the eyes had an MMT of ≤250 *μ*m posttreatment.

### 3.8. Relationship between Anatomical Response and Time Taken to Deliver the First 6 Anti-VEGF Injections

The time taken to deliver the first 6 anti-VEGF injections was analysed based on interquartile ranges to assess anatomical outcomes (i.e., achievement of a ≥20% reduction in CMT from baseline). The mean time to first 6 anti-VEGF increased with interquartile range, i.e., quartile 1, 210 ± 39.1 days; quartile 2, 328.4 ± 29.5 days; quartile 3, 471.5 ± 59.2; and quartile 4, 849.7 ± 226.4 days. Increased time to deliver the first 6 anti-VEGF was associated with a worsening of anatomical outcomes; the percentage of patients achieving a ≥20% reduction in CMT from baseline was highest in quartile 1 and lowest in quartile 4 (quartile 1, 60%; quartile 2, 47%; quartile 3, 47%; and quartile 4, 40%).

### 3.9. Foveal Contour

Individual OCTs were read and classified as “dry” where a normal foveal contour was observed in the absence of oedema. Following treatment, only 13.9% (*n* = 11/79) of eyes were classified as “dry.”

## 4. Discussion

As far as we are aware, this is the first UK retrospective study reporting “real-world” anti-VEGF use in pseudophakic eyes treated for DMO, with a view to understanding anatomical and functional outcomes, alongside anti-VEGF treatment patterns.

The time interval to receive 6 intravitreal injections of anti-VEGF was much slower than reported in RCT data for ranibizumab, aflibercept, and the DRCR.net protocol T study [12, 14, 24]. Overall, functional outcomes were poor, and treatment rates were suboptimal when compared with randomised controlled trials [12, 14, 24]. However, these findings are similar to those of previous publications using real-world data [32–38]. Recently, Kodjikian et al. [39] conducted an analysis of 63 observation studies, of which 32 included anti-VEGF, covering a total of 6,842 eyes. Among these eyes, a mean gain of +4.7 letters was observed for a mean of 5.8 injections at 15.6 months. Previously, Ciulla et al. [40] had reported real-world DMO data on more than 28 000 eyes and found a mean letter gain of 4 to 4.5 letters at 1 year with a mean of 6.4 anti-VEGF injections. Both real-world summaries trend similarly to our cohort with a mean letter gain of 1.6 letters after 6 anti-VEGF injections achieved in 9–12 months. Furthermore, a tendency to switch within the class was observed, despite the lack of evidence to support this practice. The suboptimal anatomical and functional outcomes observed in this cohort do little to build support for such practice [21]. This study highlights key challenges in delivering DMO therapy in a real-world setting with patients waiting for 77.8 days per injection for the first 6 anti-VEGF injections. The reasons are not fully explored in this study. However, it is likely related to increasing patient numbers along with insufficient capacity for clinic and injection appointments and a need for frequent administration of IVT to maintain/improve vision.

Currently approved anti-VEGF product licences specify how often patients should be reviewed after starting on a course of intravitreal therapy for DMO. These treatments normally require monthly or bimonthly reviews, depending on therapy [41, 42]. We observed that schedules are often suboptimal and patients are reviewed less frequently after the last intravitreal injection. It may be that the presence of DMO in those instances is interpreted as a recurrence rather than an insufficient response. If the window for observing treatment response is repeatedly missed, it is possible that patients become trapped in a “cycle of recurrence.” It is of course impossible to differentiate between these two scenarios in the current data set. However, without addressing the systemic reasons that led to the initial delay, it may not be helpful to represcribe anti-VEGF with the hope to review the patient in a timely manner. Furthermore, there seems to be significant variation between clinicians as to when to switch therapies in DMO [9]. In light of these considerations, and unless further resources become available in clinics, there is a need for longer-acting treatments that reduce treatment demand and the burden on health care resources. We suggest that corticosteroid treatment plus 2–3 monthly monitoring visits would have been more beneficial to this pseudophakic cohort and more sustainable for the clinic, compared to the 2–3 monthly anti-VEGF therapy they received.

The advantage of corticosteroid treatment over anti-VEGF therapy is emphasised by Kodjikian et al. in the aforementioned analysis in which 1,703 eyes, over 31 studies, received intravitreal corticosteroids (dexamethasone). These showed greater gains in VA (+9.6 letters for a mean of 1.6 injections at 10.3 months follow-up) compared with eyes in the anti-VEGF studies, especially for higher baseline VA. A factor that may have impacted the treatment of this population is a possible reluctance to consider corticosteroid treatment. Despite being pseudophakic when anti-VEGF treatment was commenced and more than 50% having had a suboptimal anatomical response (<20% reduction in CMT) after 6 injections, none of these eyes were prescribed IVT corticosteroids. This may be attributed to side effects, such as raised intraocular pressure, which, when coupled with the long duration of effect, may have influenced decision-making.

### 4.1. Limitations

Prescribing patterns for different anti-VEGF agents may have led to a situation whereby in some eyes, following initial monthly injections, there was a resolution of oedema or maximum visual acuity was achieved. Monitoring and treatment intervals might then have been extended, resulting in the longer periods taken to deliver 6 anti-VEGF injections, which were observed in this study. However, the functional and anatomical outcomes reported here suggest that this was unlikely. Furthermore, the time taken to deliver the first 6 anti-VEGF injections was analysed based on interquartile ranges to assess anatomical outcomes (i.e., achievement of a ≥20% reduction in CMT from baseline). The mean time to first 6 anti-VEGF increased with interquartile range (quartile 1, 210 ± 39.1 days and quartile 4, 849.7 ± 226.4 days), and this was associated with a worsening of anatomical outcomes (60% in quartile 1 and 40% in quartile 4).

Additionally, the overall age of this cohort is slightly higher than average for DMO patients, and although this is to be expected in a pseudophakic population, it nonetheless means a heightened likelihood of increased disease chronicity and associated deterioration of retinal health. This may have impacted the outcomes achieved.

Furthermore, measurements may differ between OCT devices, especially between different types, such as swept-source and spectral-domain OCT devices [43, 44]. In one comparison, values for CRT were found to be significantly lower for Topcon DRI OCT-1 compared to Heidelberg Spectralis [44]. Therefore, there may be discrepancies in the observed outcomes in our review, although most patients had been examined with the same machine. Consistency in the OCT device used is crucial for accurate monitoring of DMO in patients.

Finally, the known limitations with any retrospective analyses of electronic medical records apply; for example, no further information could be obtained if there were missing data points, and the quality of data was dependent on the completeness and accuracy of electronic records. It is possible that some patients' records were incomplete (i.e., additional anti-VEGF were delivered but not captured in the electronic medical record). It was not possible to cross-check all the anti-VEGF treatments recorded in Medisoft against the injection clinic logbooks.

## 5. Conclusions

In conclusion, this retrospective study shows that the anti-VEGF treatment received by DMO patients in this service was suboptimal both in the intensity of delivery and the outcomes observed. These findings confirm recent “real-world” data. Despite being candidates for second-line treatment with longer-acting intravitreal corticosteroids, there was a strong tendency for patients to be switched within the class to a second anti-VEGF agent.

These results suggest that despite anti-VEGF therapy having proven efficacy, extended periods between reviews may be responsible for the suboptimal results. Considering this, if there is no capacity to review ongoing anti-VEGF therapy in a timely manner, ophthalmologists should feel encouraged to move onto second-line intravitreal corticosteroid therapies rather than switching to another anti-VEGF.

Given the growing prevalence of DMO, there is a need to establish a shared protocol for multidisciplinary teams delivering DMO services which specifies when and how treatment response should be assessed, defines insufficient treatment response, and outlines the appropriate next steps in patients with suboptimal response to first-line therapies. This frees up anti-VEGF clinic space for those who will benefit more. Such clear protocols allow patients to benefit from the full range of NICE-approved DMO treatments available, clear pathways and processes, and workable systems to ensure timely follow-up.

## Figures and Tables

**Table 1:** Baseline demographics and ocular characteristics.

Characteristics	Mean ± SD or number of cases
Total number of eyes (*n*)	79
Age (range, years)	71 (range: 44–89)
Sex (male/female)	53/26
Eye treated (right/left)	38/41
Lens status	77^*∗*^ pseudophakic
Mean IVI received by *N* eyes	11.1 ± 4.7
Time to all injections (days)	913 ± 454.1
Total IVI count (*n*)	876
Ranibizumab	467
Aflibercept	391
Bevacizumab	19
IVTA	4 ^*∗∗*^
BRVA (logMAR)	0.55
CMT (*μ*m)	474.4
MMT (*μ*m)	496.3

^*∗*^Two cases were awaiting cataract surgery at baseline;  ^*∗∗*^four cases were excluded from the current analysis. BRVA: best-recorded visual acuity; CMT: central macular thickness; IVI: intravitreal injection; IVTA: intravitreal triamcinolone acetonide; MMT: maximal macular thickness; SD: standard deviation; VEGF: vascular endothelial growth factor.

**Table 2:** Overall rate of anti-VEGF treatment.

Anti-VEGF treatment	Anti-VEGF IVI count	Time to 6 injections (days)	Time to all injections (days)	Injection rate—first 6 (days per injection)	Injection rate—all (days per injection)
All	11.1 ± 4.7 (9)	466.9 ± 270.3 (376)	913.0 ± 454.1 (854)	77.8 ± 45.0 (62.7)	83.9 ± 35.4 (76.5)
First 6 IVI—EYLEA, *N* = 10	7.7 ± 1.3 (8)	328.4 ± 110.0 (302.5)	418.8 ± 155.3 (445.5)	54.7 ± 18.3 (50.4)	53.8 ± 17.4 (56.0)
First 6 IVI—Lucentis, *N* = 35	12.2 ± 5.2 (11)	480.5 ± 260.8 (451.0)	1094.0 ± 335.5 (1213.0)	80.1 ± 43.5 (75.2)	97.6 ± 34.6 (91.4)
First 6 IVI—mixed, *N* = 33	10.8 ± 4.2 (9)	498.0 ± 307.8 (373.0)	860.2 ± 507.3 (763.0)	83.0 ± 51.3 (62.2)	78.4 ± 34.4 (72.7)

Data are presented as mean ± SD with the median shown in parenthesis.

## Data Availability

The datasets analysed during the current study are available from the corresponding author upon reasonable request.

## Supplementary Materials

Supplementary Materials Supplemental Figure S1: maximal macular thickness at baseline and after 5 anti-VEGF injections. Supplemental Table S1: anatomical response after 5 anti-VEGF injections (defined by experiencing ≥20% reduction from baseline).
