# Supplementary material for: Retrospective Analysis of Treatment Patterns in Pseudophakic Diabetic Macular Oedema Eyes Treated with Anti-VEGF
Source: J Ophthalmol. 2021 Jul 27;2021:9967831. doi: 10.1155/2021/9967831 (PMC8337101; doi:10.1155/2021/9967831)
Supplement: Supplementary Materials — Supplemental Figure S1: maximal macular thickness at baseline and after 5 anti-VEGF injections. Supplemental Table S1: anatomical response after 5 anti-VEGF injections (defined by experiencing ≥20% reduction from baseline). [file 9967831.f1.docx]

**SUPPLEMENTARY MATERIALS**

**Supplemental Figure S1.** Maximal macular thickness at baseline and post 5 anti-VEGF injections.


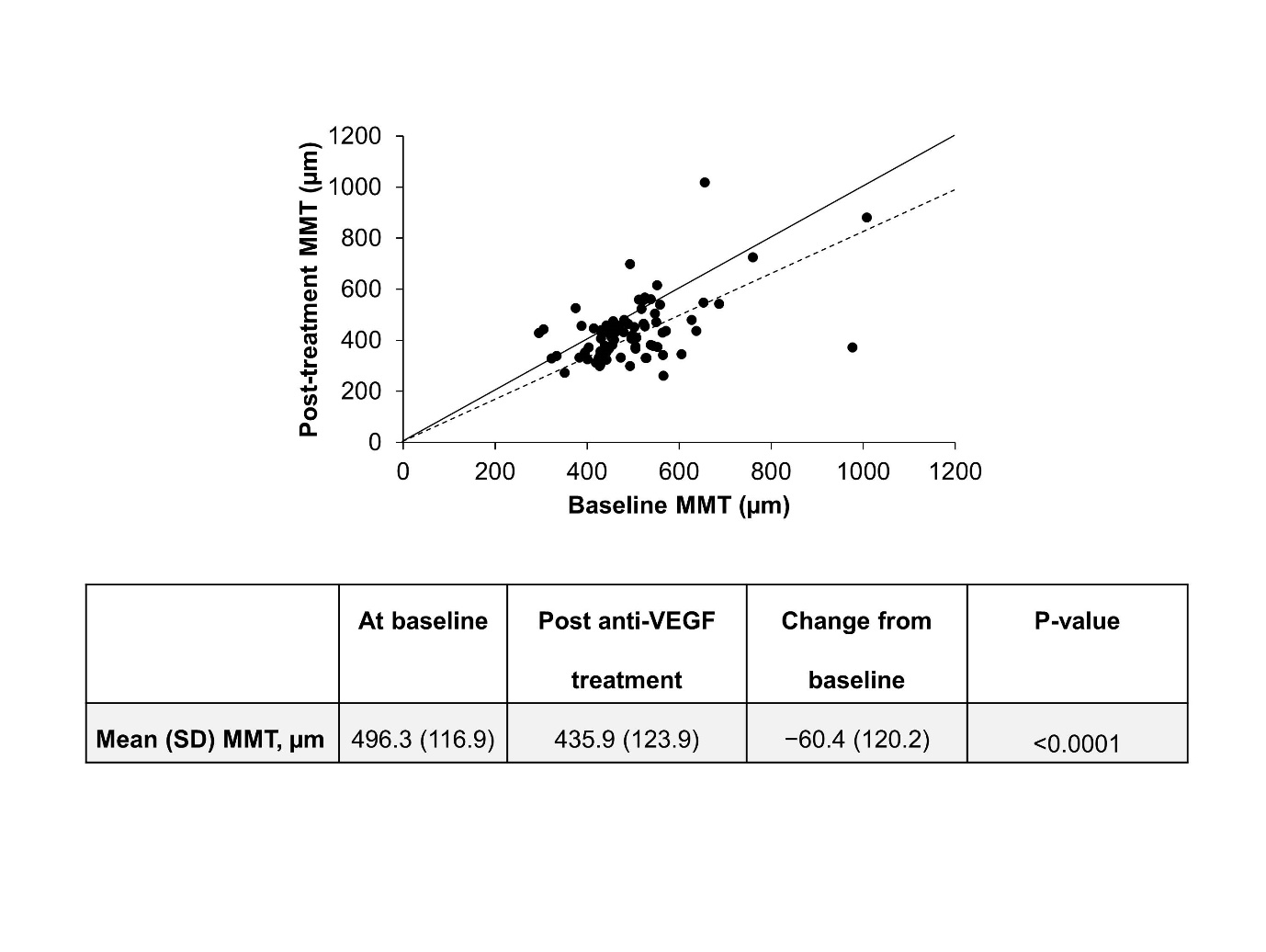


Note: The solid line represents the 45-degree line and the dashed line represents a 20% change from the 45-degree line following treatment.

**Supplemental Table S1.** Anatomical response after five anti-VEGF injections (defined by experiencing a ≥20% reduction from baseline)**.**

| **Parameter** | **Reduction of ≥50µm** | **Reduction of ≥20%** | **≤250µm** | **≤300µm** | **≤400µm** |
| --- | --- | --- | --- | --- | --- |
| CMT, N (%) | 48 (61.5) | 38 (48.7) | 5 (6.4) | 23 (29.5) | 47 (60.3) |
| MMT, N (%) | 41 (52.6) | 25 (32.1) | 0 (0.0) | 4 (5.1) | 32 (41.0) |

Notes: CMT, central macular thickness, MMT, maximal macular thickness, VEGF, vascular endothelial growth factor.
